# Lung protective effect of Ticagrelor in endotoxemia

**DOI:** 10.25122/jml-2022-0308

**Published:** 2023-06

**Authors:** Ruaa Murtada Mueen, Maytham Al-Juaifari, Munther Abosaooda, Heider Qassam, Najah Rayish Hadi

**Affiliations:** 1 Department of Pharmacology & Therapeutics, Faculty of Medicine, University of Kufa, Kufa, Iraq; 2 KMG Klinikum Güstrow, Clinic for Trauma Surgery, Spinal Surgery and Orthopedics, Güstrow, Germany; 3 College of Pharmacy, Islamic University, Najaf, Iraq; 4 Department of Pharmacology, Faculty of Medicine, University of Kufa, Iraq

**Keywords:** lung protective effect, Ticagrelor, endotoxemia

## Abstract

Sepsis is a life-threatening organ dysfunction caused by a dysregulated host response to infection. This study aimed to investigate the potential protective effect of the lungs in sepsis by modulating inflammatory and oxidative stress markers. Twenty-four adult male Swiss-albino mice, aged 8-12 weeks and weighing 20-30 g, were divided into four equal groups (n=6): sham (laparotomy only), CLP (laparotomy plus cecal ligation and puncture), vehicle (DMSO administered one hour before CLP), and Ticagrelor (50 mg/kg IP administered one hour before CLP). Tissue levels of pro-inflammatory and oxidative stress markers in the lung were assessed using ELISA. F2 isoprostane levels were significantly higher in the sepsis group (p<0.05) compared to the sham group, while Ticagrelor significantly decreased the inflammatory and oxidative stress markers compared to the sepsis group. All mice in the sepsis group had considerable (p=0.05) lung tissue damage, but Ticagrelor considerably decreased lung tissue injury (p=0.05). Furthermore, Ticagrelor was found to reduce tissue cytokine levels of the lung (IL-1, TNF a, IL-6, F2 isoprostane, GPR 17, MIF) in male mice during CLP-induced polymicrobial sepsis by modulation of pro-inflammatory and oxidative stress cascade signaling pathways.

## INTRODUCTION

Despite advances in supportive treatment, sepsis remains a serious public health problem, imposing substantial medical, social, and financial burdens on healthcare systems. With an annual mortality rate of more than one in four individuals worldwide, sepsis continues to be a leading cause of death [1]. Sepsis is described as life-threatening organ dysfunction induced by an unbalanced host response to infection [2] and can develop from any infection, including bacterial, parasite, or viral sources such as malaria or COVID-19 [3]. The lung is one of the initial organs to be affected by the systemic inflammatory response of sepsis, which results in alveolar or capillary cell injury, diffuse pulmonary edema, and exudation, followed by respiratory failure, that is acute and noncardiogenic pulmonary edema [4]. The discovery of key products, such as cytokines that can induce sepsis-like clinical symptoms in laboratory animals has driven the search for specific agents capable of neutralizing or mitigating these detrimental effects [5].

Inflammatory cytokines play a crucial role in mediating the innate immune response to infections, with monocytes producing elevated levels of pro-inflammatory cytokines such as interleukin-6 (IL-6) and IL-1β [6]. High concentrations of these pro-inflammatory cytokines can contribute to the aggravation of endotoxin shock [7]. IL-6 has been associated with tissue damage from inflammation, while IL-1β has been implicated in lung inflammation [8].

Ticagrelor, a potent antiplatelet agent, acts as a direct-acting, reversible inhibitor of platelet purinergic receptors (P2Y12) [9]. As the first oral P2Y12 antagonist, Ticagrelor exhibits fast absorption and prevents platelet aggregation. Consequently, it is considered first-line therapy for acute coronary syndrome (ACS). The therapeutic effects of Ticagrelor largely depend on the primary molecule and its metabolites. In this study, we aimed to investigate the potential protective effects of Ticagrelor on sepsis by modulating inflammatory and oxidative stress markers.

## MATERIAL AND METHODS

### Study location

This study was conducted at the Department of Pharmacology and Therapeutics, Faculty of Medicine, University of Kufa, and the Middle Euphrates Unit for Cancer Research. Adult male Swiss albino mice, aged 8-12 weeks and weighing 21-28 grams, were obtained from the Resources for Animals Centre of the College of Science, University of Kufa. The mice were housed in the university's animal facility, maintained at a temperature of 25°C and a humidity of 60-65%, with a 12-hour light and dark cycle.

### Group allocation

A total of 24 mice were randomly assigned to four groups, with six mice in each group:

1. Sham group: Mice in this group underwent laparotomy surgery without cecal ligation and puncture (CLP).
2. Sepsis (CLP) group: Mice in this group underwent cecal ligation and puncture to induce sepsis.
3. Vehicle group: Mice in this group received an intraperitoneal injection of an equivalent volume of dimethyl sulfoxide (DMSO) one hour before CLP treatment.
4. Ticagrelor pre-treated group: Mice in this group received an intraperitoneal injection of Ticagrelor at 50 mg/kg one hour before CLP treatment.

### Experimental procedure

Mice were anesthetized with 100 mg/kg ketamine and 10 mg/kg xylazine administered intraperitoneally. A 1.5 cm midline incision was made to perform an abdominal laparotomy, exposing the cecum. The cecum was ligated below the ileocecal valve and punctured twice using a G-22 needle. The abdominal incision was sutured with 5/0 surgical sutures [10]. Mice were monitored every four hours for 24 hours to observe signs of illness and were then returned to their cages with unrestricted access to food and water [10, 11].

### Drug preparation

Ticagrelor was obtained from Med. Chem. Express in the United States and formulated as a 100 mg/ml solution in DMSO and SBE. Ticagrelor was administered intraperitoneally at 50 mg/kg [12]. The lungs of the mice were removed 24 hours after CLP, and tissue samples were divided in half. One half was stored at -80 degrees Celsius for subsequent ELISA analysis, while the remaining samples were fixed in 10% formalin for histological evaluation.

### Tissue sample preparation for histopathology

To remove red blood cells and clots, lung tissues were washed with a cold isotonic sodium chloride solution (0.9%). The tissues were then fixed in 10% formalin and processed into paraffin tissue blocks. After fixation, the specimens were dehydrated in a series of ethanol concentrations (70%, 80%, 90%, and 100%) and cleared with organic xylene solvent to remove residual alcohol. Subsequently, the tissues were embedded in paraffin wax. The histopathological examination was carried out at magnifications ranging from X100 to X400 [13] and evaluated using the proportion of tissue damage as follows:

- Score 0: Normal architecture with no damage
- Score 1: Mild damage (25% damage)
- Score 2: Moderate damage (25-50% damage)
- Score 3: Severe damage (50-75% damage)
- Score 4: Highly severe damage (75-100% damage)

### Statistical Analysis

Statistical analysis was performed using SPSS version 26. Data are presented as Mean±SEM. Multiple group comparisons were analyzed using analysis of variance (ANOVA), followed by a post-hoc test with Bonferroni correction. The Kruskal-Wallis test was used to determine the statistical significance of differences in mean scores for histological abnormalities in lung tissue between groups. A p-value of 0.05 was considered statistically significant.

## RESULTS

### Ticagrelor decreased inflammatory IL-6, IL-1β, and TNFα markers levels

In the sepsis group (control), TNFα lung concentration was significantly higher (p<0.05) compared to the sham group, while the difference between the sepsis and vehicle groups was minimal.

**Figure 1:**
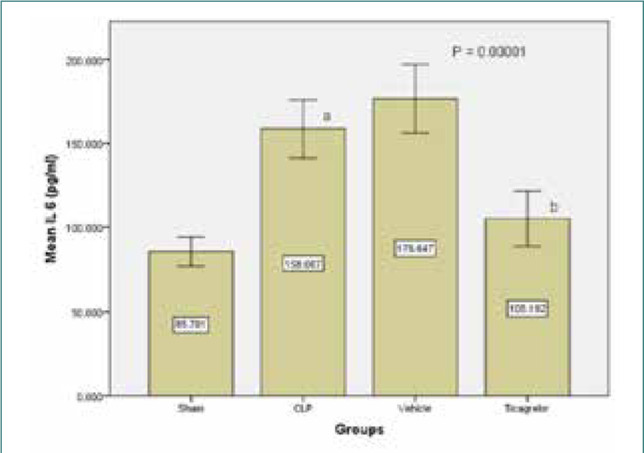
Mean lung tissue levels of TNFα (ng/L) ± SEM in the study groups; sham versus sepsis (p-value=0.00001), Ticagrelor versus sepsis and vehicle (p-value=0.0001)

**Figure 2:**
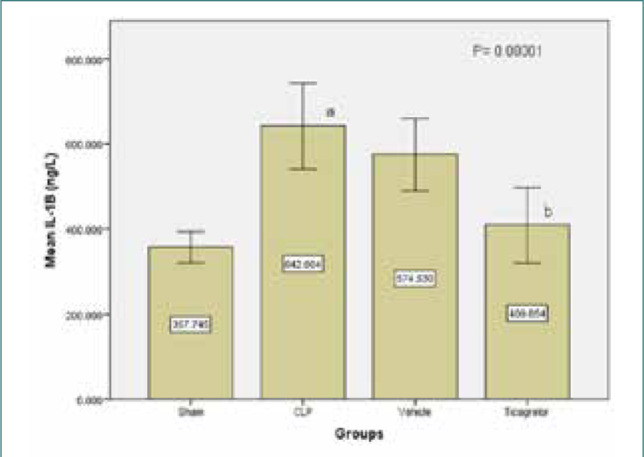
Mean lung tissue levels of IL1β (ng/L) ± SEM in the study groups; sham vs. sepsis (p-value=0.00001), Ticagrelor vs. sepsis and vehicle (p-value=0.0001)

**Figure 3:**
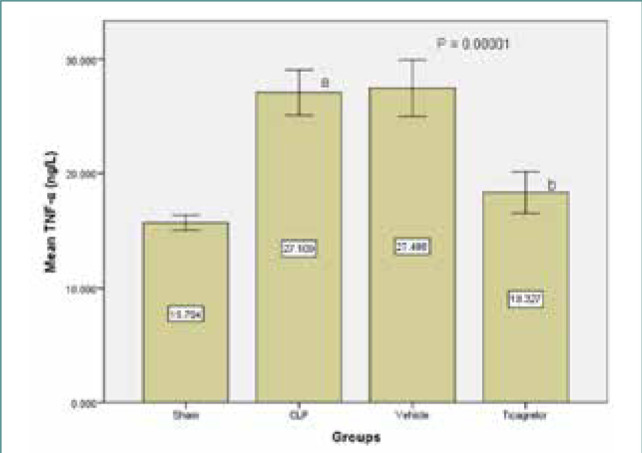
Mean lung tissue level of TNFα (ng/L) ± SEM in the study groups; sham vs. sepsis (p-value=0.00001), Ticagrelor vs. sepsis and vehicle (p-value=0.0001)

Ticagrelor treatment resulted in lower tissue levels of TNFα in the lung compared to the sepsis and vehicle groups (Figure 1). Similarly, levels of IL-6 and IL-1β in the lung tissue were significantly higher (p<0.05) in the sepsis group compared to the sham group. However, the Ticagrelor-treated group showed significantly decreased expression of the inflammatory markers IL-6 and IL-1β compared to the sepsis group (Figures 2 and 3).

### Ticagrelor decreased GPR17 marker expression

**Figure 4:**
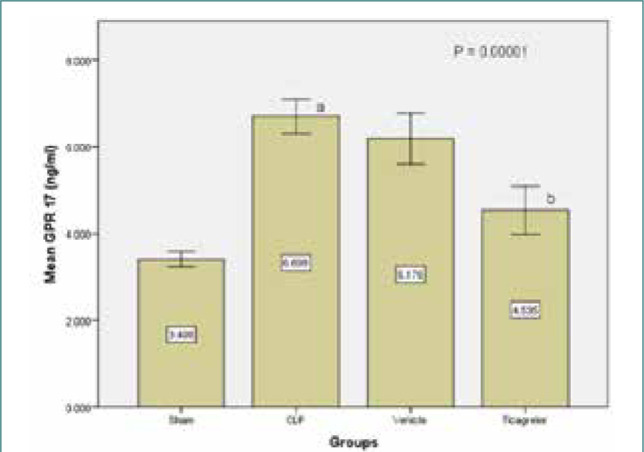
Mean lung tissue level of GPR17 (ng/ml) ± SEM in the study groups; sham vs. sepsis (p-value=0.00001), Ticagrelor vs. sepsis and vehicle (p-value=0.0001)

The levels of GPR17 in the lung tissue were significantly higher (p<0.05) in the sepsis group compared to the sham group. However, the Ticagrelor-treated group showed significantly decreased GPR17 expression compared to the sepsis group (Figure 4).

### Ticagrelor reduced F2-isoprostane levels in the lung tissue

**Figure 5:**
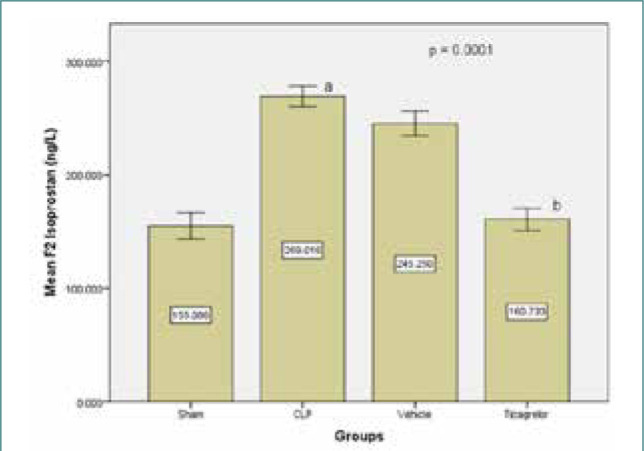
Mean lung level of F2 isoprostane (ng/L) ± SEM in the study groups; sham vs. sepsis (p-value=0.00001), Ticagrelor vs. sepsis and vehicle (p-value=0.0001)

The sepsis group had a significant increase in F2-isoprostane levels in the lung tissue compared to the sham group. Ticagrelor pretreatment decreased lung tissue levels of F2-isoprostane compared to the sepsis group (Figure 5).

### Ticagrelor decreases lung tissue MIF level

**Figure 6:**
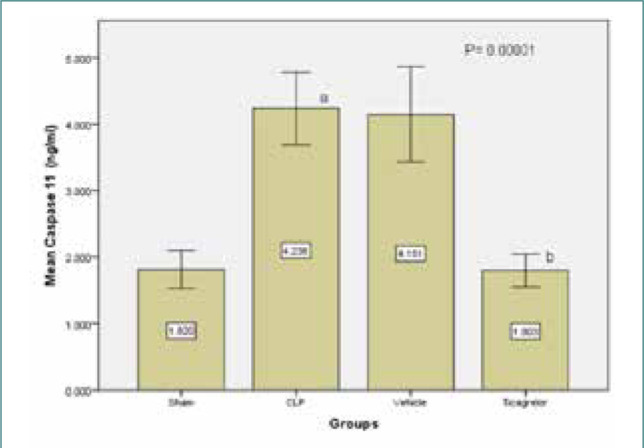
Mean caspase-11 (ng/L) ± SEM lung tissue level of the study groups; sham vs. sepsis group (p-value=0.00001), Ticagrelor vs. sepsis and vehicle group (p-value=0.0001)

The sepsis group showed a significant increase in MIF levels in the lung tissue compared to the sham group. Ticagrelor pretreatment led to decreased lung tissue levels of MIF compared to the sepsis group (Figure 6).

### Ticagrelor decreased caspase-11 levels in lung tissue

**Figure 7:**
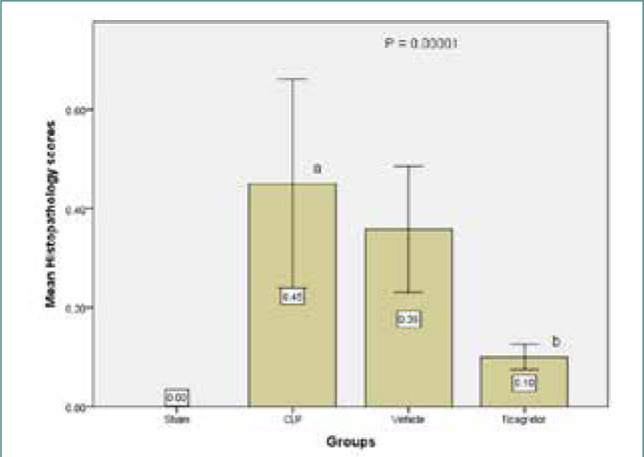
Lung tissue histopathology results of the study groups; sham vs. sepsis and vehicle group (p-value=0.0001), Ticagrelor vs. sepsis group (p-value=0.0001), Ticagrelor vs. sepsis group (p-value=0.0001)

Compared to the control group, the sepsis group exhibited a significant increase in caspase-11 levels in the lung tissue. Ticagrelor pretreatment decreased caspase-11 levels in the lung tissue compared to the sepsis group (Figure 7).

### Ticagrelor minimized lung injury

#### Sham group

**Figure 8:**
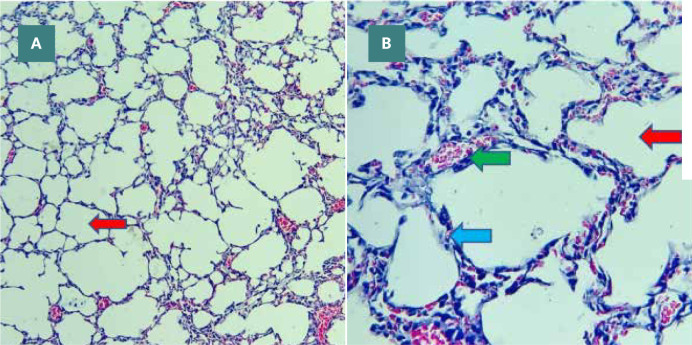
Sham group lung tissue showing alveoli (red arrows), inter-alveolar septa (blue arrow), and capillaries (green arrow) H&E, A X100, B X400

Histological examination of the Ticagrelor-treated group showed mild architectural alterations compared to the sham group. This group showed mild damage in histopathological grading from normal lung tissue, as shown in Figure 8 (A-B). The lung tissue in the sham group had normal architecture with no signs of erythrocyte leakage, leukocyte infiltration, inflammation, edema, or hyperemia.

#### Sepsis group

**Figure 9:**
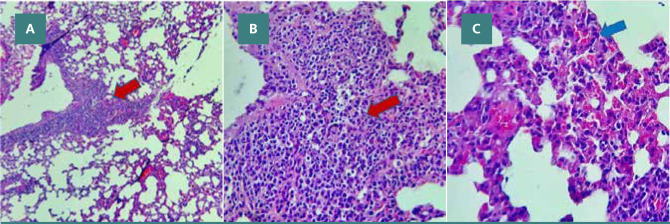
CLP group lung tissue with severe inflammation (red arrows) involving 70% of the examined lung tissue, intra-alveolar and perivascular inflammation with hyperemia (blue arrows), H&E A X40, B X100, C X400

**Figure 10:**
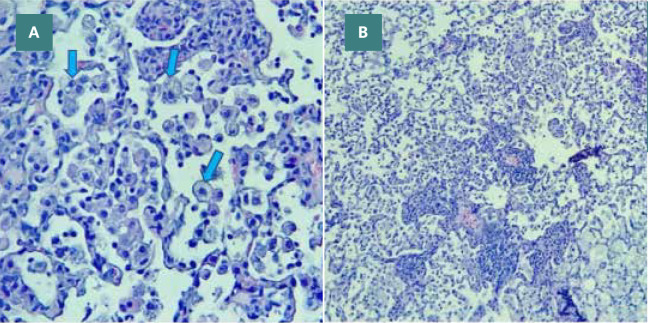
CLP group lung tissue with severe inflammation involving 80% of the examined lung tissue, intra-alveolar inflammation with abundant macrophages (arrows), and hyperemia, H&E A X40, B X400

In contrast, the lung tissue from the sepsis group exhibited significant signs of lung injury, including hyperemia, heavy perivascular inflammation characterized by the presence of lymphocytes and plasma cells, interstitial edema, and extravasation of red blood cells. In addition, the alveoli showed the presence of neutrophils and macrophages. These histopathological changes indicating lung injury are depicted in Figure 9 (A-C) and Figure 10 (A-B).

#### Vehicle group

**Figure 11:**
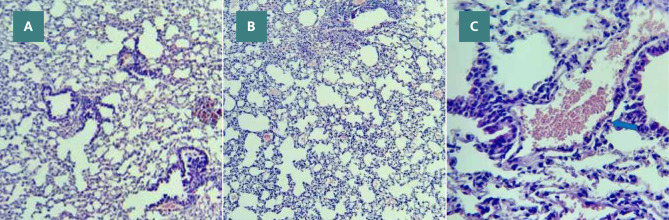
Vehicle group lung tissue with moderate degree inflammation involving 30% of the examined lung tissue, accumulation of hemosiderin-laden macrophages (red arrows), and congestion (blue arrow) H&E A X40, B X400, C X400

The lung tissue from the vehicle group, similar to the sepsis group, exhibited lung injury characterized by congestion, heavy perivascular inflammation, interstitial edema, and extravasation of red blood cells. Moreover, neutrophils and macrophages were observed within the alveoli. These histopathological changes indicating lung injury are depicted in Figure 11 (A-C). The vehicle group had a moderate degree of damage in terms of histopathological grading when compared to normal lung tissue.

#### Ticagrelor treated group

The Ticagrelor-treated group showed mild alterations to the architecture compared to the sham group. This group showed mild damage in histopathological grading from normal lung tissue.

## DISCUSSION

### Sepsis effect on pro-inflammatory cytokines (IL1β, IL-6, and TNF-α)

The study found significantly higher levels of pro-inflammatory cytokines (TNFa, IL1B, and IL-6) in the lung tissue of the sepsis and vehicle groups compared to the sham group. These findings are consistent with previous studies [11] and support the notion that sepsis induces increased blood levels of IL-1β and IL-6[11, 12]. Pro-inflammatory cytokine levels can potentially have an impact on microcirculation [13]. The main injury associated with sepsis-induced acute respiratory distress syndrome (ARDS) is the hyperpermeability of the pulmonary microvasculature, leading to the accumulation of plasma exudate in the alveoli [14]. Additionally, sepsis causes alveolar edema due to increased exudate in the alveolar spaces and the loss of alveolar epithelial cells through apoptosis and necrosis. Early and acute episodes of ARDS are characterized by widespread damage.

### Effect of Ticagrelor on proinflammatory cytokines (IL1β, IL-6, TNF-α)

Compared to the sepsis and vehicle groups, the Ticagrelor-pretreated group had significantly decreased lung tissue levels of pro-inflammatory cytokines (IL-6, IL1B, and TNF-α). Similar results were reported by another study [15], which showed reduced blood concentrations of TNF-α and IL-1 β with Ticagrelor treatment. Additionally, Tang *et al*. [16] reported a decrease in IL-1β levels, while Liverani *et al*. [17] observed a decrease in plasma TNF-α and IL-6 levels with Ticagrelor treatment. The study looked at the role of P2Y12 in controlling sepsis-induced increases in plasma cytokine levels (tumor necrosis factor-, interleukin-6, and macrophage inflammatory protein-1β). Ticagrelor-treated and P2Y12-deficient mice exhibited increased plasma levels of each cytokine during sepsis compared to sham controls [18]. In the PLATO study, Ticagrelor was associated with reduced lung infections and mortality following pulmonary infections and sepsis compared to Clopidogrel. This result may be attributed to the ability of Ticagrelor to enhance neutrophil chemotaxis and phagocytosis [19].

### Sepsis effect on F2 isoprostane level

The present study found significantly higher levels of F2 isoprostane in the lung tissue of the sepsis group compared to the sham group. Increased oxidative stress and reactive oxygen species production play a critical role in initiating and sustaining the inflammatory response. These findings align with previous studies [19], which suggest that oxidative stress contributes to mitochondrial and endothelial dysfunction in sepsis and highlight the potential of oxidative stress biomarkers for sepsis diagnosis and prognosis.

### Ticagrelor effect on F2 isoprostane

The study demonstrated that the Ticagrelor-treated group significantly reduced lung tissue levels of F2 isoprostane compared to the sepsis and vehicle groups. Apart from its action on platelets, Ticagrelor also inhibits adenosine resorption [20]. Adenosine has been shown to limit platelet activation [21] and reduce reactive oxygen species production in tissue environments [22]. This mechanism may explain how Ticagrelor affects oxidative stress biomarkers.

### Effect of sepsis on the level of caspase-11

The study found significantly increased tissue levels of caspase-11, which are associated with pyroptosis, in the lung tissue of the sepsis and vehicle groups compared to the sham group. These findings are consistent with previous studies [23-25] that reported elevated caspase-11 expression in CLP-induced ALI models and following LPS injection. Caspase-11 is an endogenous receptor for cytosolic LPS recognition, leading to pyroptosis [26]. Notably, caspase-11 knockout mice are resistant to LPS-induced septic shock [26-28], but the metabolic regulation of the caspase-11 inflammasome remains unclear [28].

### Effect of Ticagrelor on the level of caspase-11

The present study showed that the Ticagrelor-treated group exhibited a greater decrease in the tissue lung levels of caspase-11 compared to the sepsis and vehicle groups. To the best of our knowledge, no previous studies have investigated the effect of Ticagrelor on caspase-11 levels.

### Effect of sepsis on the level of GPR17

The study found significantly increased tissue levels of GPR17 in the lung tissue of the sepsis and vehicle groups compared to the sham group. These findings align with a previous study [29] that also reported an increase in GPR17 receptor levels. Modulating GPR17 has shown the potential to improve inflammatory responses and injury, as evidenced by the suppression of microglial activation and inflammation in an ischemic stroke model [30] and the protection against myocardial fibrosis caused by cardiac ischemia [31].

### Effect of Ticagrelor on the level of GPR 17

The study demonstrated that the Ticagrelor-treated group exhibited a greater decrease in the tissue lung levels of GPR17 compared to the sepsis and vehicle groups. Previous reviews [32] have indicated that Ticagrelor is one of the few medications that inhibit the GPR17 receptor.

### Effect of sepsis on the level of MIF

The study found significantly higher tissue levels of MIF in the lung tissue of the sepsis and vehicle groups compared to the sham group. Our findings were comparable with the outcome of a previous study [33]. MIF is a pro-inflammatory cytokine found in macrophages, vascular endothelial cells, tissue epithelial cells, and cancer cells [34] and influences innate immunity via TLR4 by recruiting immune cells to inflammatory locations, activating inflammatory pathways, and inducing immune cell differentiation.

### Effect of Ticagrelor on the level of MIF

The study showed that the Ticagrelor-treated group exhibited a greater decrease in the tissue lung levels of MIF compared to the sepsis and vehicle groups. To the best of our knowledge, no previous studies have investigated the effect of Ticagrelor on MIF levels.

## CONCLUSION

All mice in the sepsis group had considerable (p<0.05) lung tissue damage, but Ticagrelor considerably decreased lung tissue injury (p<0.05). Ticagrelor reduced tissue cytokine levels (IL-1, TNF a, IL-6, F2 isoprostane, GPR17, MIF) in the lung tissue of male mice during CLP-induced polymicrobial sepsis by modulation of pro-inflammatory and oxidative stress cascade signaling pathways.
